# Tandem imine formation *via* auto-hydrogen transfer from alcohols to nitro compounds catalyzed by a nanomagnetically recyclable copper catalyst under solvent-free conditions†

**DOI:** 10.1039/d1ra02347k

**Published:** 2021-05-26

**Authors:** Sara Sobhani, Hadis Hosseini Moghadam, Seyed Ruhollah Derakhshan, José Miguel Sansano

**Affiliations:** Department of Chemistry, College of Sciences, University of Birjand Birjand Iran ssobhani@birjand.ac.ir sobhanisara@yahoo.com; Departamento de Química Orgánica, Facultad de Ciencias, Centro de Innovación en Química Avanzada (ORFEO-CINQA), Instituto de Síntesis Orgánica (ISO), Universidad de Alicante Apdo. 99 03080-Alicante Spain

## Abstract

A direct imination reaction was developed by tandem reaction of alcohols and nitro compounds in the presence of Cu-isatin Schiff base-γ-Fe_2_O_3_ as a nanomagnetically recyclable catalyst under solvent-free conditions. By this method, various imines were prepared in good to high yields from one-pot reaction of various alcohols (primary aromatic and aliphatic) and nitro compounds (aromatic and aliphatic) *via* an auto-hydrogen transfer reaction. Use of an inexpensive and easily reusable catalyst, without requiring any additives or excess amounts of benzyl alcohol as the reaction solvent are the other advantages of this method. This catalytic system has the merits of cost effectiveness, environmental benignity, excellent recyclability and good reproducibility.

## Abstract 2

A direct imination reaction was developed by tandem reaction of alcohols and nitro compounds in the presence of Cu-isatin Schiff base-γ-Fe_2_O_3_ as a nanomagnetically recyclable catalyst.

## Graphical abstract

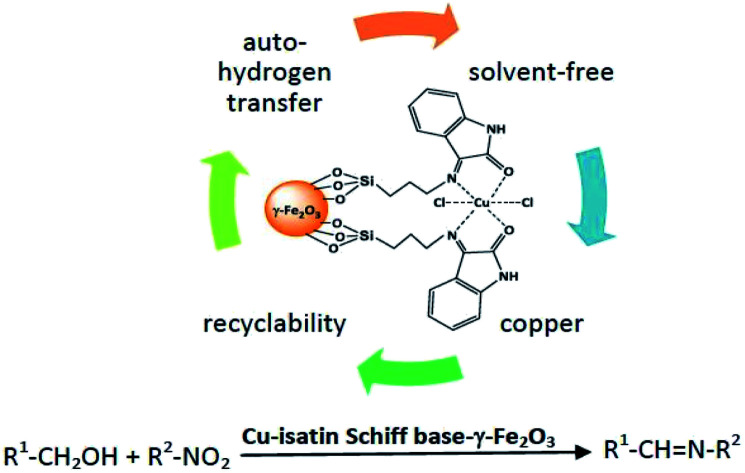

## Introduction

Imines are crucial intermediates in the synthesis of biologically active nitrogen compounds, such as β-lactams, dyes, fragrances, pharmaceuticals, fungicides, and agricultural chemicals.^1^ The C=N bond in imines has electrophilic properties and is widely used in organic transformations such as reduction, addition, cyclization, and aziridination reactions.^2^ Traditionally, imines are produced from the condensation of primary amines with carbonyl compounds.^3^ In recent years, much attention has been paid to tandem processes as an attractive synthetic concept for improving overall process efficiency and reducing waste production by converting simple starting materials into more complex products in a single reaction vessel.^4^ In this regard, tandem synthesis of imines from alcohols and nitro compounds is a more advantageous method than traditional procedures, because alcohols are much more stable and readily available starting materials.^5^ The process involves selective oxidation of benzyl alcohol to benzaldehyde followed by the coupling reaction of amines with benzaldehyde.^6^ From the standpoint of sustainable development, an atom-economic, “green” and operationally convenient method, the synthesis of imines from nitro compounds is highly desirable.^7^ In this regard, the borrowing-hydrogen methodology (auto-hydrogen transfer), which transfers hydrogen from readily available alcohols to nitro compounds to produce amines as well as valuable aldehydes, provides a promising alternative to the existing method for the synthesis of imines.^8^ Some complexes and compounds derived from transition metals of the second and third rows, such as ruthenium,^9^ palladium,^10^ silver^11^ iridium^12^ and gold^13^ have been introduced as promoters of this strategy of the imine synthesis. However, the toxicities, pricing, and stability of these metals prohibit their general use for industrial purposes.^14^ Surprisingly, there is only one report on the use of copper for imines formation *via* auto-hydrogen transfer,^15^ although copper is a low cost, easily available and relatively environmentally benign metal. This reported method suffers from severe problems related to the use of a toxic organic solvent, excess amount of the base, high temperature, and requiring prolonged reaction time (3 days).

**Scheme 1:**
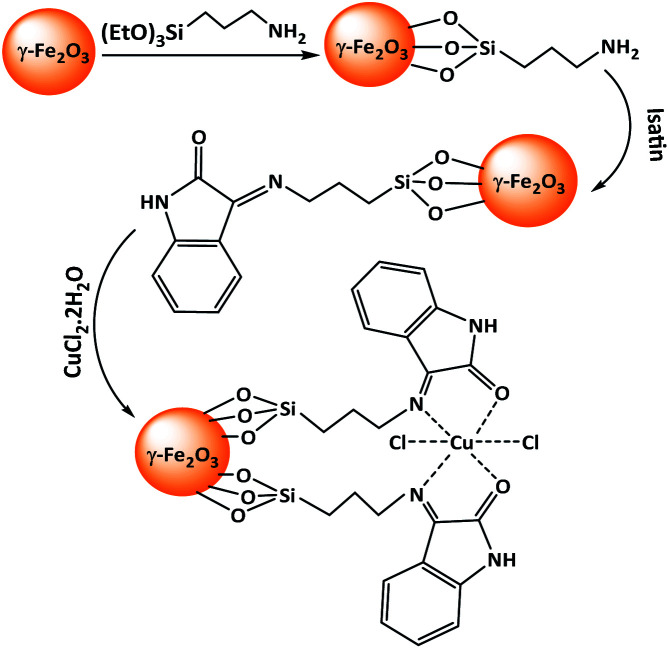
Preparation of Cu-isatin Schiff base-γ-Fe_2_O_3_.

In continuation of our efforts on the introduction of new heterogeneous catalysts for organic transformations,^16^ we have recently synthesized Cu-isatin Schiff base-γ-Fe_2_O_3_ (Scheme 1) and used it as a new heterogeneous catalyst for the synthesis of bis(indolyl)methanes and bis(pyrazolyl)methanes.^17^ Herein, in this paper, we have expanded the scope of application of this catalyst for the tandem imine formation by auto-hydrogen transfer from alcohols to nitro compounds under solvent-free conditions.

## Result and discussion

Cu-isatin Schiff base-γ-Fe_2_O_3_ as a nanomagnetically heterogeneous catalyst was synthesized according to our previously reported method.^16^ Cu-isatin Schiff base-γ-Fe_2_O_3_ as a nanomagnetically heterogeneous catalyst, was synthesized by functionalization of γ-Fe_2_O_3_ with 3-aminopropyltriethoxysilane and then the reaction with isatin to produce isatin Schiff base-γ-Fe_2_O_3_ followed by the reaction with dissolving CuCl_2_·2H_2_O in methanol (Scheme 1).

The catalyst was characterized by a series of techniques such as FT-IR, XRD, TGA, TEM, SEM, VSM, ICP and elemental analysis.

**Fig. 1:**
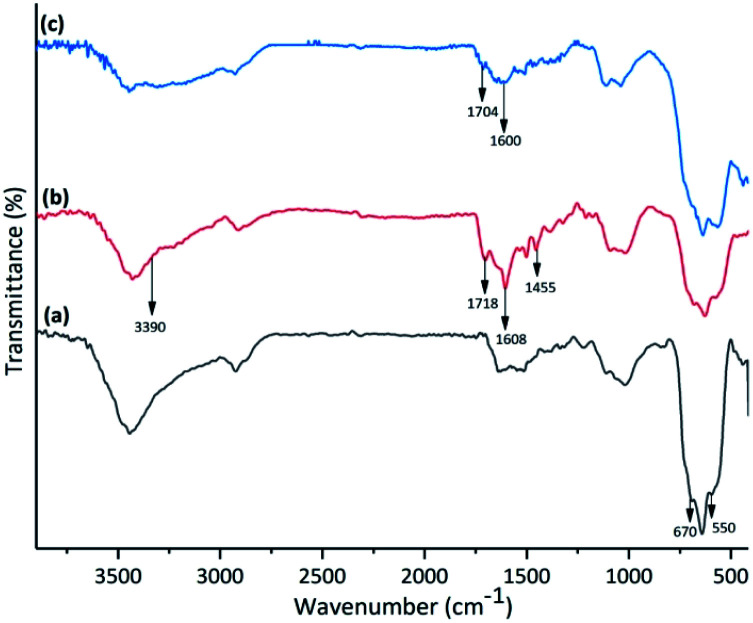
FT-IR spectra of (a) amino-functionalized γ-Fe_2_O_3_, (b) isatin Schiff base-γ-Fe_2_O_3_ and (c) Cu-isatin Schiff base-γ-Fe_2_O_3_.

The FT-IR spectra of (a) amino-functionalized γ-Fe_2_O_3_, (b) isatin Schiff base-γ-Fe_2_O_3_ and Cu-isatin Schiff base-γ-Fe_2_O_3_ are exhibited in Fig. 1. The FT-IR spectrum of amino-functionalized γ-Fe_2_O_3_ exhibited a broad band at around 550–670 cm^−1^ due to the stretching vibrations of Fe–O. The appeared peaks at 1085, 3438, 3480 and around 2869–2906 cm^−1^ in the FT-IR spectrum of amino-functionalized γ-Fe_2_O_3_ are related to C–N, N–H and C–H stretching modes of the alkyl chain, respectively. N–H bending was observed at 1631 cm^−1^ (Fig. 1a). New bands at 1455, 1608 and 1718 cm^−1^ in the FT-IR spectrum of isatin Schiff base-γ-Fe_2_O_3_ (Fig. 1b) refer to the C=C, C=N and C=O stretching vibrations, respectively. These bands proved that isatin has been successfully reacted with amino-functionalized γ-Fe_2_O_3_. N–H stretching band of the amide group in isatin overlapped with the broad O–H band, which was found at 3390 cm^−1^. In the FT-IR spectra of Cu-isatin Schiff base-γ-Fe_2_O_3_ (Fig. 1c), the C=N and C=O stretching frequencies were shifted to the lower wave numbers (1600, 1704 cm^−1^), which showed the successful coordination of nitrogen and oxygen to the metal center.

**Fig. 2:**
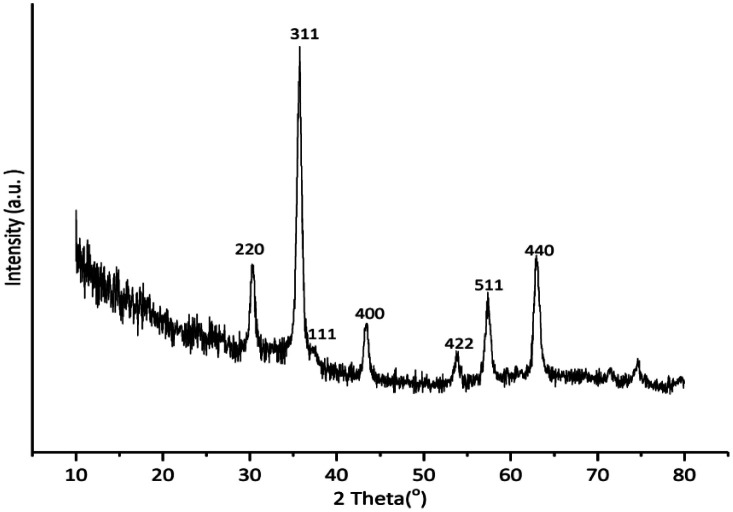
XRD pattern of Cu-isatin Schiff base-γ-Fe_2_O_3_.

As presented in Fig. 2, the reflection planes of (2 2 0), (3 1 1), (1 1 1), (4 0 0), (4 2 2), (5 1 1) and (4 4 0) at 2*θ* = 30.3°, 35.7°, 39.9°, 43.4°, 53.8°, 57.4° and 63.0° were readily recognized in the XRD pattern of Cu-isatin Schiff base-γ-Fe_2_O_3_. These characteristic peaks matched with those of standard γ-Fe_2_O_3_ (JCPDS file no. 04-0755). The observed diffraction peaks was indicated that γ-Fe_2_O_3_ mostly exists in face-centered cubic structure. In addition, the diffraction plane of (111) at 2*θ* = 37.4° in the XRD pattern of Cu-isatin Schiff base-γ-Fe_2_O_3_ is ascribed to Cu.

**Fig. 3:**
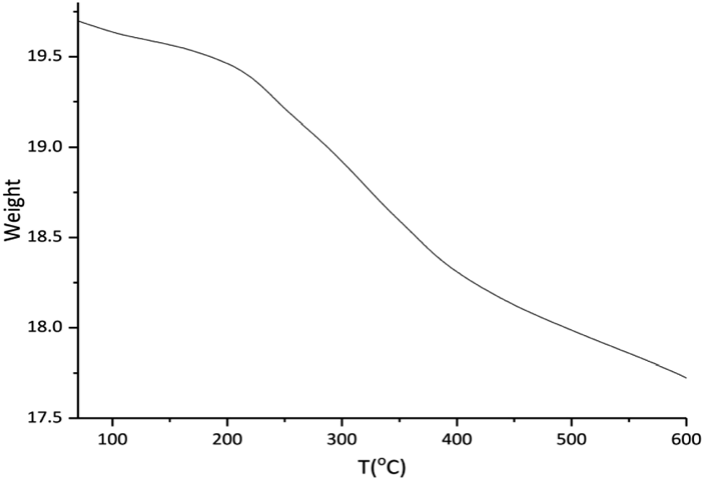
TGA diagram of Cu-isatin Schiff base-γ-Fe_2_O_3_.

The thermogravimetric analysis (TGA) of Cu-isatin Schiff base-γ-Fe_2_O_3_ was used to determine the thermal stability and content of organic functional groups on the surface of magnetic nano particles (Fig. 3). TG curve of the catalyst showed the weight loss at around 182 °C, which was related to the adsorbed water molecules on the support. The organic parts were decomposed completely in the temperature range of 200–600 °C.

The metal content of the complex was determined by ICP-OES, and showed a value of 0.11 mmol g^−1^. Elemental analysis showed that the loading of isatin-Schiff base on γ-Fe_2_O_3_, was 0.31 mmol g^−1^ based on the nitrogen and carbon amounts (0.86% and 3.95%, respectively).

**Fig. 4:**
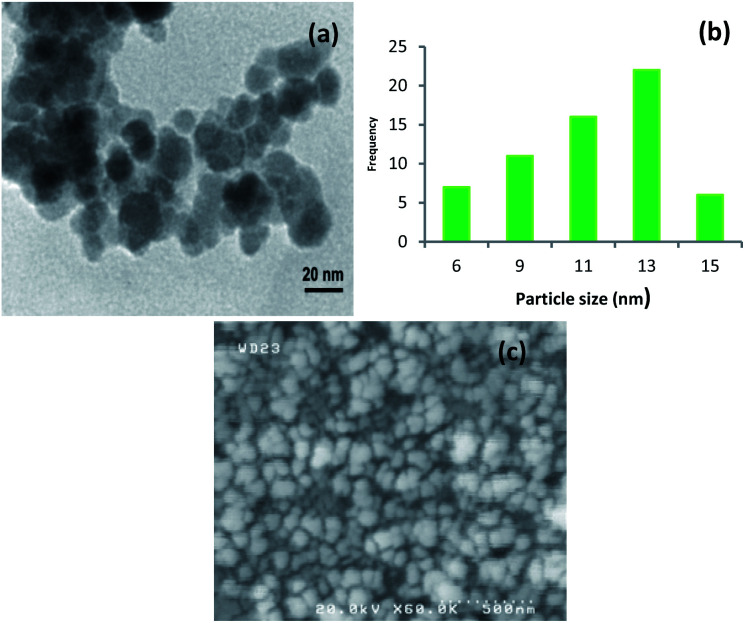
(a) TEM image of Cu-isatin Schiff base-γ-Fe_2_O_3_ and (b) particle size distribution histogram of Cu-isatin Schiff base-γ-Fe_2_O_3_. (c) SEM image of Cu-isatin Schiff base-γ-Fe_2_O_3_.

The size and morphology of the synthesized catalyst were characterized using TEM and SEM (Fig. 4). The TEM image is clearly showed that Cu-isatin Schiff base-γ-Fe_2_O_3_ exhibits spherical morphology with relatively good monodispersity (Fig. 4a). The particle size distribution of Cu-isatin Schiff base-γ-Fe_2_O_3_ was evaluated using TEM and showed that the average diameter of the particles was 13 nm (Fig. 4b). The SEM image analysis indicates that the-γ-Fe_2_O_3_ nanoparticles are spherical in shape (Fig. 4c).

**Fig. 5:**
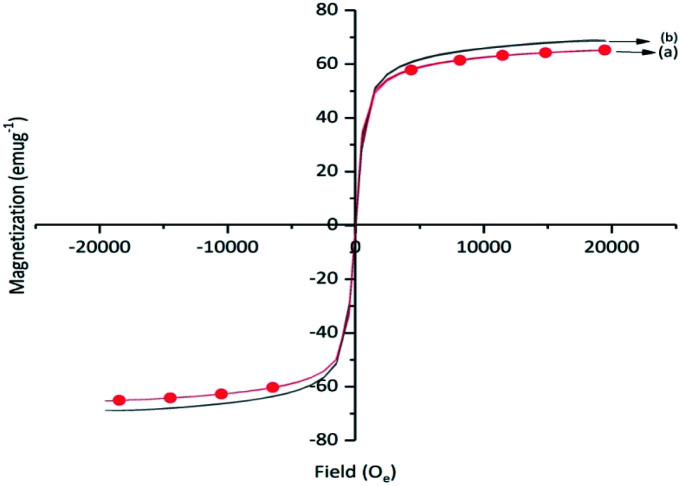
Magnetization curves of (a) Cu-isatin Schiff base-γ-Fe_2_O_3_ and (b) γ-Fe_2_O_3_.

The saturation magnetization values for γ-Fe_2_O_3_ and Cu-isatin Schiff base-γ-Fe_2_O_3_ were 68.9 and 65.2 emu g^−1^, respectively (Fig. 5). A slight decrease of the saturation magnetization of Cu-isatin Schiff base-γ-Fe_2_O_3_ was due to the immobilization of Cu complex on the surface of γ-Fe_2_O_3_.

**Fig. 6:**
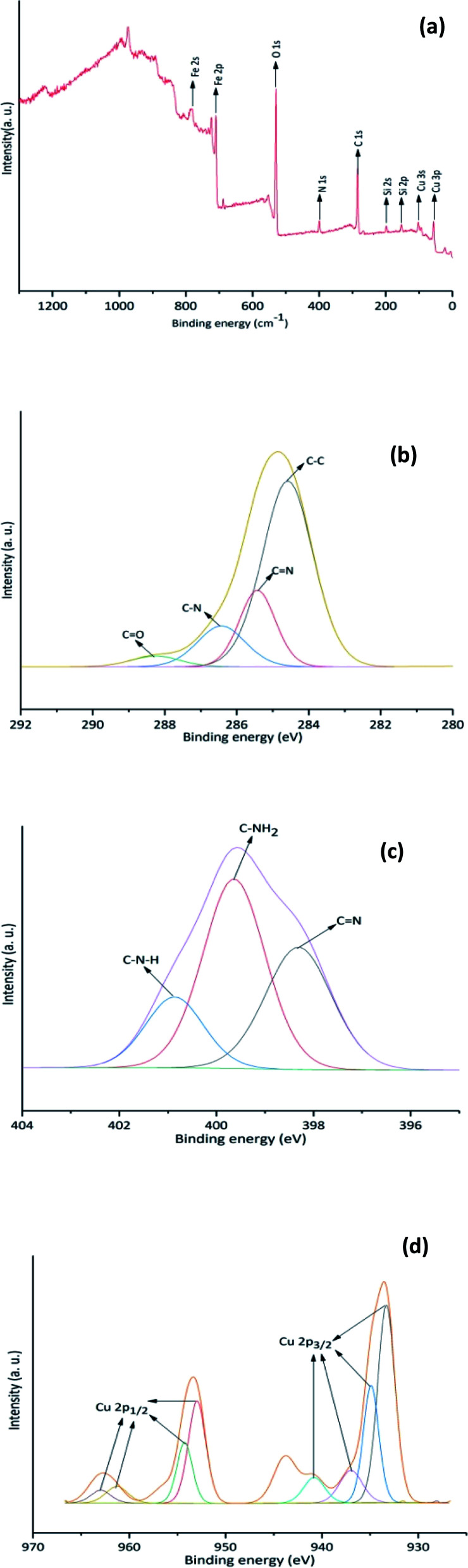
(a) XPS patterns of the Cu-isatin Schiff base-γ-Fe_2_O_3_, (b) C (1s), (c) N (1s), (d) Cu.

Cu-isatin Schiff base-γ-Fe_2_O_3_ was also analyzed by XPS spectroscopy (Fig. 6). The observed characteristic peaks in the XPS elemental survey are attributed to carbon (C 1s), nitrogen (N 1s), oxygen (O 1s), iron, silicon and copper (Fig. 6a).^18^ The C 1s spectrum (Fig. 6b) showed binding energies of 284.5 (C–C), 285.4 (C=N), 286.4 (C–N) and 288.3 eV (C=O).^19^ Furthermore, high resolution XPS of N 1s region confirmed the presence of N–H, imine and –NH_2_ by revealing three peaks at 400.8, 399.6 and 398.3 eV, respectively (Fig. 6c).^20^ In the XPS spectrum of Cu (Fig. 6d), the presence of binding energies at 933.3 and 940.8 eV (Cu 2p_3/2_) along with the binding energies at 952.9 and 963.1 eV (Cu 2p_1/2_) could be ascribed to Cu with (I) oxidation state. The peaks centered at 934.9, 936.9 (Cu 2p_3/2_) and 954.2, 961.3 eV (Cu 2p_1/2_) are attributed to the existence of Cu(ii) in the catalyst.^21^

After catalyst characterization, its catalytic activity was studied in the tandem imines formation by auto-hydrogen transfer from alcohols to nitro compounds. At first, to find out the best reaction conditions, a set of factors including base, solvent, temperature and the amount of the catalyst were screened in the reaction of benzyl alcohol and nitrobenzene as the model reaction. The study of the influence of the base, as well as its absence, showed that KOH rendered the best results (Table 1, entry 4). The solvent dependency of the model reaction was investigated (Table 1, entries 7–13) and found that the reaction proceeded efficiently under solvent-free conditions as green and sustainable reaction conditions. In an effort to optimize the reaction temperature, temperatures below 100 °C were found to minimize the imines formation (Table 1, entries 14 and 15). Screening the amount of the catalyst showed that decreasing the catalyst amount from 2 to 1 mol% led to a negligible decrease in the yield of the product (Table 1, entry 16). Poorer activity was obtained by further lowering the catalyst amount (Table 1, entry 17). To show the role of the catalyst, the reaction was performed in the absence of the catalyst and also in the presence of CuCl_2_ and isatin Schiff base-γ-Fe_2_O_3_ as a copper less analogue of the catalyst (Table 1, entries 18–20). It was found that the desired product was obtained in low yield after 24 h. These findings indicated that a desirable activation of copper was occurred by complexation in the catalyst. The reaction of benzyl alcohol was studied in the presence of the catalyst under optimized reaction conditions (Table 1, entry 21). The results showed that benzyl alcohol was oxidized and produced benzaldehyde in 60% yield after 10 h. The reaction of nitrobenzene in the presence of the catalyst was also studied and any product was not obtained (Table 1, entry 22). This should be attributed to the lack of benzyl alcohol as a driving force for subsequent reduction of nitro groups. Performing the model reaction under O_2_ and Ar atmosphere proceeded with no change in the product yields (Table 1, entries 22 and 23) compared with the optimal conditions (Table 1, entry 16), which showed that alcohol dehydrogenized without requiring specific atmospheric conditions.

**Table 1:** Optimization of the tandem imine formation *via* auto-hydrogen transfer from benzyl alcohol to nitrobenzene

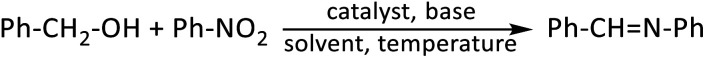						
Entry^a^	Catalyst (mol%)	Solvent	Base	Temperature (°C)	Time (h)	Isolated yield (%)
1	2	—	K_2_CO_3_	100	5	42
2	2	—	Et_3_N	100	24	Trace
3	2	—	Na_2_CO_3_	100	24	35
4	2	—	KOH	100	9	95
5	2	—	NaOH	100	9	86
6	2	—	—	100	6	30
7	2	EtOH	KOH	65	3	22
8	2	H_2_O	KOH	80	24	0
9	2	Acetonitrile	KOH	68	24	Trace
10	2	Benzyl alcohol	KOH	100	7	81
11	2	*n*-Hexane	KOH	50	5	35
12	2	Toluene	KOH	90	3	32
13	2	Ethyl acetate	KOH	60	24	Trace
14	2	—	KOH	r.t.	24	Trace
15	2	—	KOH	65	7	56
16	1	—	KOH	100	10	92
17	0.5	—	KOH	100	6	63
18	0	—	KOH	100	24	21
19^b^	1	—	KOH	100	24	43
20^c^	1	—	KOH	100	24	30
21^d^	1	—	KOH	100	10	0^e^
22^f^	1	—	KOH	100	10	0
22^g^	1	—	KOH	100	10	90
23^h^	1	—	KOH	100	12	87

a Reaction conditions: nitrobenzene (0.5 mmol, except for entry 21), benzyl alcohol (0.5 mmol, except for entry 22), base (0.5 mmol, except for entry 6), solvent (3 mL, entries 7–13).

b CuCl_2_·2H_2_O was used as a catalyst.

c Isatin Schiff base-γ-Fe_2_O_3_ as a catalyst.

d Without nitrobenzene.

e Benzaldehyde was obtained as the only product in 60% yield.

f Without benzyl alcohol.

g Under O_2_.

h Under Ar.

With an optimized catalytic system in hand (Table 1), we set out to probe versatility of the present method in the direct imine synthesis of various alcohols and nitro compounds by auto-hydrogen transfer strategy. As depicted in Table 2, the reaction of various nitrobenzenes and benzyl alcohols catalyzed by Cu-isatin Schiff base-γ-Fe_2_O_3_ gave expected imines (a–m) in good to high yields (Table 2, entries 1–13). The reaction of benzyl alcohol with nitroethane as an aliphatic compound and also the reaction of nitrobenzene with 1-butanol as an aliphatic alcohol proceeded well under optimized reaction conditions (Table 2, entries 14 and 15). The imine products in the current study have widespread usages in the synthesis of biologically active compounds with antibacterial and antifungal activities.^22^

**Table 2:** Tandem imines formation *via* auto-hydrogen transfer from alcohols to nitro compounds catalyzed by Cu-isatin Schiff base-γ Fe_2_O_3_

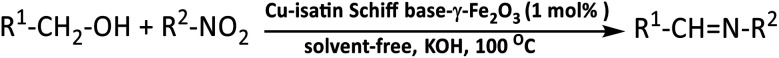					
Entry^a^	R^1^	R^2^	Product	Time (h)	Yield (%)
1	C_6_H_5_	C_6_H_5_	a	10	92
2	C_6_H_5_	4-OH–C_6_H_4_	b	8	90
3	C_6_H_5_	4-OMe–C_6_H_4_	c	8	84
4	C_6_H_5_	4-Me–C_6_H_4_	d	9	87
5	C_6_H_5_	4-Cl–C_6_H_4_	e	12	71
6	4-Me–C_6_H_4_	C_6_H_5_	f	13	91
7	4-Me–C_6_H_4_	4-Cl–C_6_H_4_	g	12	68
8	4-Me–C_6_H_4_	4-OMe–C_6_H_4_	h	10	85
9	4-Me–C_6_H_4_	4-Me–C_6_H_4_	i	12	83
10	4-OMe–C_6_H_4_	C_6_H_5_	j	12	75
11	4-OMe–C_6_H_4_	4-Cl–C_6_H_4_	k	13	72
12	4-OMe–C_6_H_4_	4-OMe–C_6_H_4_	l	11	84
13	4-OMe–C_6_H_4_	4-Me–C_6_H_4_	m	14	70
14	C_6_H_5_	CH_3_–CH_2_–	n	10	72
15	CH_3_–CH_3_–CH_2_–	C_6_H_5_	o	12	85

a Reaction conditions: nitro compound (1 mmol), alcohol (1 mmol), KOH (1 mmol), solvent-free, catalyst (1 mol%), 100 °C. Melting points of the solid products were compared with the reported ones in the ESI (Table S1).

**Fig. 7:**
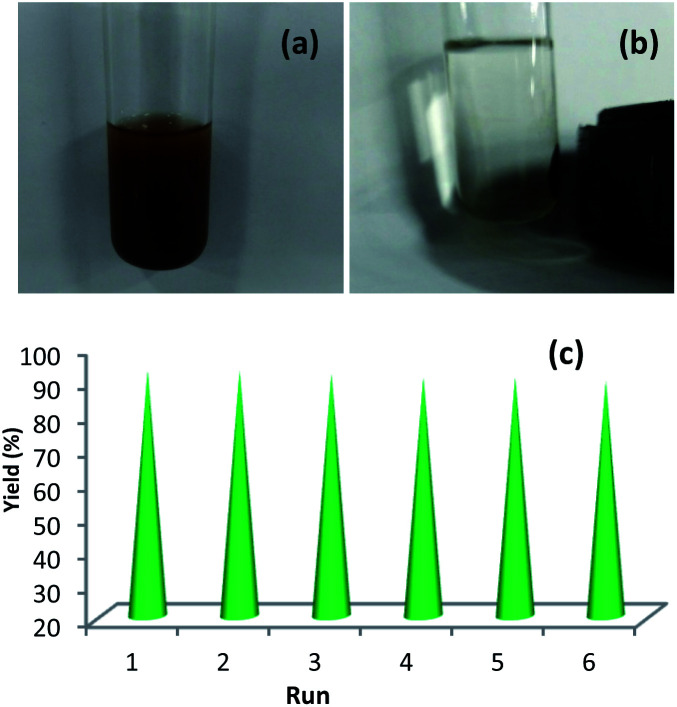
(a) Reaction mixture after adding EtOAc, (b) isolation of the catalyst by an external magnet (c) reusability of Cu-isatin Schiff base-γ-Fe_2_O_3_ in the reaction of nitrobenzene and benzyl alcohol at 100 °C in 10 h.

The recyclability of Cu-isatin Schiff base-γ-Fe_2_O_3_ was investigated in a model reaction of benzyl alcohol and nitrobenzene in the auto-hydrogen transfer reaction, under optimized reaction conditions. EtOAc was added to the reaction mixture after 10 h (Fig. 7a). The catalyst was isolated by an external magnet (Fig. 7b), washed with EtOAc and EtOH (2 × 10 mL) and dried under vacuum. The catalyst was successfully recycled for six times. Loss of the catalytic activity was not considerably observed for Cu-isatin Schiff base-γ-Fe_2_O_3_ in these reactions (Fig. 7c).

**Fig. 8:**
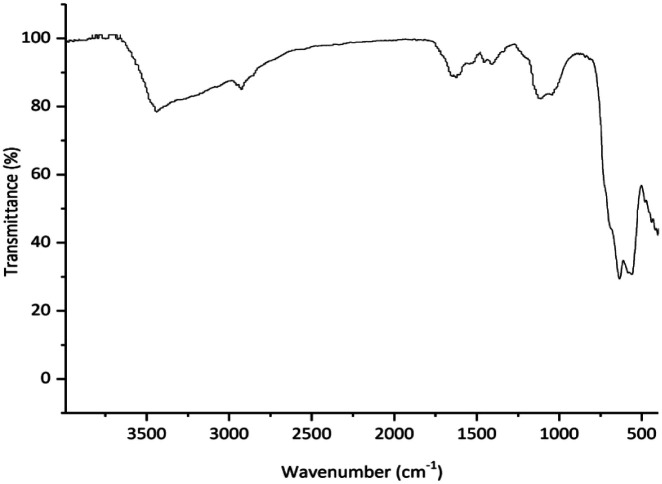
FT-IR spectrum of the Cu-isatin Schiff base-γ-Fe_2_O_3_ after six times reuse.

Comparison of FT-IR of the reused catalyst (Fig. 8) with the freshly prepared one (Fig. 1c) indicated that any significant changes in the chemical structure of the catalyst was not observed.

**Fig. 9:**
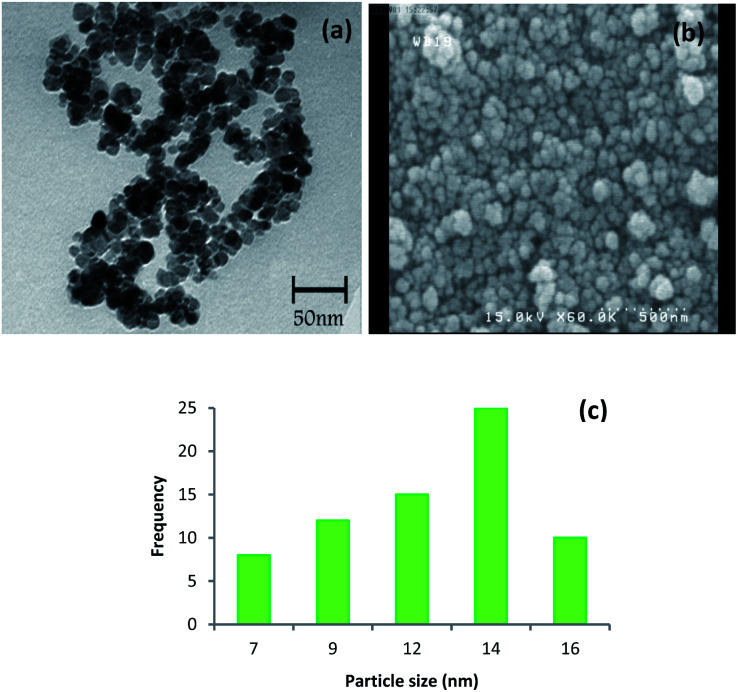
(a) TEM image, (b) FE-SEM image and (c) size distribution of the Cu-isatin Schiff base-γ-Fe_2_O_3_ after six times reuse.

Moreover, TEM and FE-SEM images illustrated that the nanoparticles were still spherical in shape even after six times reuse (Fig. 9a and b) and the mean diameter size of the recycled catalyst was 14 nm (Fig. 9c).

To study the synthetic applications and practical activity of this protocol, the reaction of benzyl alcohol (50 mmol) and nitrobenzene (50 mmol) was assessed under the optimized reaction conditions and the desired product was isolated in 90% yield after 13 h.

**Scheme 2:**
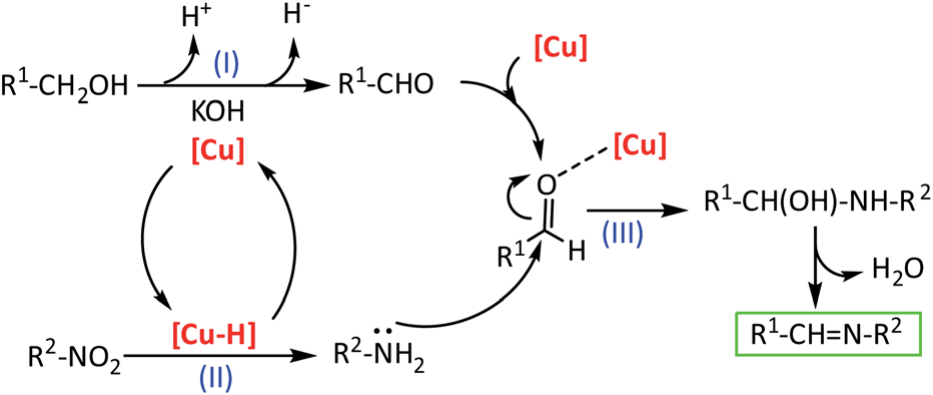
A plausible mechanism for the tandem reaction catalyzed by Cu-isatin Schiff base-γ-Fe_2_O_3_.

Based on our experiment results and previous literature reports,^10*b*,12,13,14,26^ a reaction mechanism to rationalize the direct imines formation from the reaction of nitroarenes with alcohols was depicted in Scheme 2. First, the alcohol is dehydrogenated to aldehyde in the presence of Cu-isatin Schiff base-γ-Fe_2_O_3_ as a catalyst promoted by base, releasing proton and hydride (step I). Simultaneously, the hydride is transferred to the copper complex to give a copper–hydride complex. This copper–hydride complex reduces the nitro group to amine *via N*-phenylhydroxylamine as a well-known intermediate (step II).^23^ In the next step, the nucleophilic attack of the amine to the activated aldehyde by the copper catalyst followed by water elimination gives the imine product (step III). In this mechanism the copper catalyst promoted both the hydrogen transfer from the alcohol to the nitro compounds and also the imine formation from the *in situ* produced aldehyde and amine. To investigate the mechanism in detail, the reaction of *N*-phenylhydroxylamine and benzyl alcohol under optimized reaction conditions was employed. The imine was obtained in 80% yield after 8 h. The reaction of benzaldehyde with aniline was also studied and the desired imine was produced in 93% yield after 6 h.

**Scheme 3:**
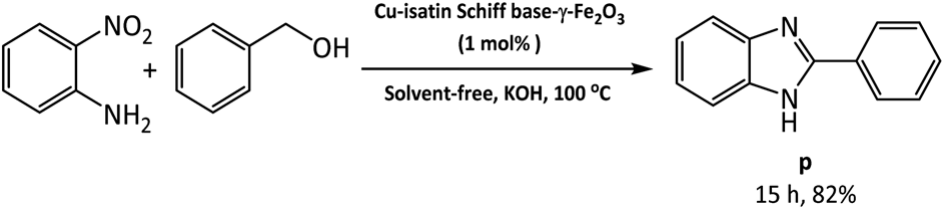
Tandem reaction of 2-nitroaniline with benzyl alcohol under the optimized reaction conditions.

The applicability of this method was also examined for the one-pot reaction of 2-nitroaniline with benzyl alcohol under the optimized reaction conditions at the last part of our studies. Benzimidazole (p) was produced in 82% yields by auto-hydrogen transfer reaction in the presence of Cu-isatin Schiff base-γ-Fe_2_O_3_ (Scheme 3).

To show the merits of the present protocol for the synthesis of imines, we have compared the catalytic activity of Cu-isatin Schiff base-γ-Fe_2_O_3_ with those of some reported catalysts in the auto-hydrogen transfer reactions (Table 3). As depicted in Table 3, Cu-isatin Schiff base-γ-Fe_2_O_3_ is the most effective catalyst for the reaction of benzyl alcohols and nitrobenzenes. The reported synthetic routes have certain limitations such as requiring high temperature, long reaction times, expensive catalysts, additives, large amount of the catalyst, bimetallic catalysts, especial atmospheric conditions, toxic organic solvents and most importantly use of excess amounts of benzyl alcohol as the reaction solvent. More importantly, our catalyst is a magnetic heterogeneous catalyst and can be easily separated from the reaction mixture using an external magnet.

**Table 3:** Comparison of catalytic activity of Cu-isatin Schiff base-γ-Fe_2_O_3_ with some reported catalysts for the auto-hydrogen transfer reactions of alcohols with nitro compounds

Entry (ref.)	Catalyst (mol%)	Reaction conditions	Time (h)	Yield (%)
1 (ref. 10*a*)	Pd/HT^a^ (2)	Toluene, 130 °C	24	35–93
2 (ref. 11)	Ag-MCP-1^b^ (25 mg)	Toluene, K_2_CO_3_, glycerol, 120 °C	12	70–99
3 (ref. 24)	Rh/Au (0.5)	Benzyl alcohol (solvent), Cs_2_CO_3_, 100 °C	22	57
4 (ref. 25)	Co–N–C/CNT@AC^c^ (0.4 g)	Solvent free, N_2_ atmosphere, 160 °C	18–42	12–100
5 (ref. 26)	Ir^III^–Au^I^ heterodimetallic complex (1)	Benzyl alcohol (solvent), Cs_2_CO_3_, 100 °C, aerobic condition	15–22	4–99
6 (ref. 14)	CoO_*X*_@NC-800^d^ (10)	Toluene, ^*t*^BuOK, 120 °C	15	57–87
7 (ref. 12)	Ir–Pd heterodimetallic catalyst (2)	Benzyl alcohol (solvent), Cs_2_CO_3_, 110 °C	3–20	16–92
8 (ref. 15)	CuO–Fe_3_O_4_ (1.3)	Toluene, NaOH, 130 °C	3 d	58–84
9 (ref. 10*b*)	Pd/DNA (2.9)	Water, LiOH·H_2_O, 50 °C, N_2_ balloon	24	51–95
10 (ref. 13)	Au/Ag–Mo-NR^e^ (40 mg)	Toluene, K_2_CO_3_, glycerol, Ar, 120 °C	24	60–98
11 (ref. 9)	RuCl_3_ (3)	K_2_CO_3_, glycerol, N_2_ atmosphere	24	72–99
12 (This work)	Cu-isatin Schiff base-γ-Fe_2_O_3_ (1)	Solvent-free, KOH, 100 °C	8–14	65–92

a Hydrotalcite.

b Polyacrylic acid.

c Co, N and C composite encapsulated carbon nanotube grown *in situ* on the surface of activated carbon.

d Cobalt nanoparticles modified with N-doped hierarchical porous carbon derived from biomass.

e Au/Ag–Mo nano-rods.

## Experimental

### General procedure for the tandem synthesis of imine from alcohols and nitro compounds

Cu-isatin Schiff base-γ-Fe_2_O_3_ (1 mol%) was added to a mixture of alcohol (1 mmol), nitro compound (1 mmol) and KOH (1 mmol) and the resulting mixture was stirred at 100 °C for an appropriate time (Table 2). After cooling the reaction mixture to room temperature, EtOAc (5 mL) was added to the reaction mixture. The catalyst was separated by an external magnet, washed with EtOAc (2 × 10 mL) and EtOH (2 × 10 mL), dried in vacuum and reused. The solvent of the combined organic layer was evaporated under vacuum. Pure products were obtained by recrystallization in EtOH or by column chromatography eluted with *n*-hexane : EtOAc (5 : 1 or 2 : 1). ^1^H NMR spectra of the products (a, c, d, e, g, k and p) have been presented in the ESI.†

## Conclusion

In summary, in this paper, an efficient method for the synthesis of imines by tandem reaction of various alcohols (primary aromatic and aliphatic) and nitro compounds (aromatic and aliphatic) was developed *via* auto-hydrogen transfer reaction, catalyzed by Cu-isatin Schiff base-γ-Fe_2_O_3_ as a nanomagnetically reusable and inexpensive catalyst under solvent-free conditions. Good to high yields of imines were achieved under mild reaction conditions without requiring any additive. After EtOAc was added to the reaction mixture, the catalyst was easily isolated by using an external magnet and reused successfully for six runs without any significant changes on the chemical structure of the catalyst. This catalytic system had the merits of cost effectiveness, environmental benignity, excellent recyclability and good reproducibility for the direct synthesis of imines from alcohols and nitro compounds without requiring high temperature, long reaction times, expensive catalysts, additives, large amounts of the catalyst, bimetallic catalysts, especial atmospheric conditions, hydrogen gas, toxic organic solvents and most importantly use of excess amounts of benzyl alcohol as the reaction solvent.

## Conflicts of interest

There are no conflicts to declare.

## Supplementary Material

RA-011-D1RA02347K-s001
