# Supplementary material for: Tandem imine formation via auto-hydrogen transfer from alcohols to nitro compounds catalyzed by a nanomagnetically recyclable copper catalyst under solvent-free conditions
Source: RSC Adv. 2021 May 26;11(31):19121–7. doi: 10.1039/d1ra02347k (PMC9033619; doi:10.1039/d1ra02347k)
Supplement: RA-011-D1RA02347K-s001 [file RA-011-D1RA02347K-s001.pdf]

Electronic Supplementary Material (ESI) for RSC Advances.
This journal is © The Royal Society of Chemistry 2021

Tandem imines formation *via* auto-hydrogen transfer from alcohols to nitro compounds catalyzed by a nanomagnetically recyclable copper catalyst under solvent-free conditions

Sara Sobhani ,*[a] Hadis Hosseini Moghadam,[a] Seyed Ruhollah Derakhshan,[a] José Miguel Sansano[b]

General information

Chemicals were purchased from Merck Chemical Company. NMR spectra were recorded in ppm in $CDCl_3$ on a Bruker Avance DPX-300 instrument using TMS as internal standard. The purity of the products and the progress of the reactions were accomplished by TLC on silica-gel polygram SILG/UV254 plates. The morphology of the products was determined by using Hitachi Japan, model s4160 Scanning Electron Microscopy (SEM) at accelerating voltage of 15 KV. TEM analysis was performed using TEM microscope (philips CM30). Power X-ray diffraction (XRD) was performed on a Bruker D8-advance X-ray diffractometer or on a X'Pert Pro MPD diffractometer with Cu K_α (λ = 0.154 nm) radiation. Thermo gravimetric analysis (TGA) was performed using a Shimadzu thermo gravimetric analyzer (TG-50). FT-IR spectra were recorded on a JASCO FT-IR 460 plus spectrophotometer. The content of Cu in the catalyst was detemined by OPTIMA 7300DV ICP analyzer. Elemental analysis was carried out on a Costech 4010 CHN elemental analyzer. Room temperature magnetization isotherms were obtained using a vibrating sample magnetometer (VSM, LakeShore 7400). XPS analyses were performed using a VG-Microtech Multilab 3000 spectrometer, equipped with an Al anode. The deconvolution of spectra was carried out by using Gaussian–Lorentzian curves.

[a.] *Address: Department of Chemistry, College of Sciences, University of Birjand, Birjand, Iran, email: ssobhani@birjand.ac.ir, sobhanisara@yahoo.com.*

[b.] *Departamento de Qu´ımica Org´anica, Facultad de Ciencias, Centro de Innovaci´on en Qu´ımica Avanzada (ORFEO-CINQA) and Instituto de S´ıntesis Org´anica (ISO), Universidad de Alicante, Apdo. 99, 03080-Alicante, Spain.*

Synthesis of Cu-isatin Schiff base-γ-Fe_2O_3[1]

The synthesized amino-functionalized γ-Fe_2O_3[2] (3 g) was sonicated in absolute ethanol (50 mL) for 30 min. Isatin (1.9 g, 13 mmol) was added slowly to the sonicated mixture and stirred at 80 °C for 24 h. The resulting mixture was cooled to room temperature. The solid was separated by an external magnet, washed with EtOH (3 × 10 mL) and Et_2O (3 × 10 mL) and dried at 50 °C in oven under vacuum to give isatin Schiff base-γ-Fe_2O_3. Isatin Schiff base-γ-Fe_2O_3 (3 g) was added to a solution of $CuCl_2.2H_2O$ (0.21 g) in MeOH (30 mL). The reaction mixture was stirred at room temperature for 24 h. After stirring, the solid was separated by an external magnet, and washed with MeOH (3 × 10 mL) and Et_2O (3 × 10 mL). It was then dried in an oven at 80 °C overnight to give Cu-isatin Schiff base-γ-Fe_2O_3.

Table S1. Melting point of the products

Entry	Compound	Obtained m.p. (°C)	Reported m.p. (°C)[ref]
1	**a**	49-50	50.1-52.2[3]
2	**b**	193-195	192-194[4]
3	**c**	67-69	68.3-70.5[5]
4	**d**	51-53	52[6]
5	**e**	59-61	58-60[7]
6	**f**	46-48	45-47[7]
7	**g**	118-120	117-119[7]
8	**h**	80-82	81-83[8]
9	**i**	51-53	52-54[8]
10	**j**	66-68	65-67[8]
11	**k**	91-93	92[9]
12	**l**	141-143	142.2-144.4[5]
13	**m**	92-94	93-94[10]
14	**n**	-[a]	-
15	**o**	-[a]	-
16	**p**	292-294	295[11]

[a]Brown liquid

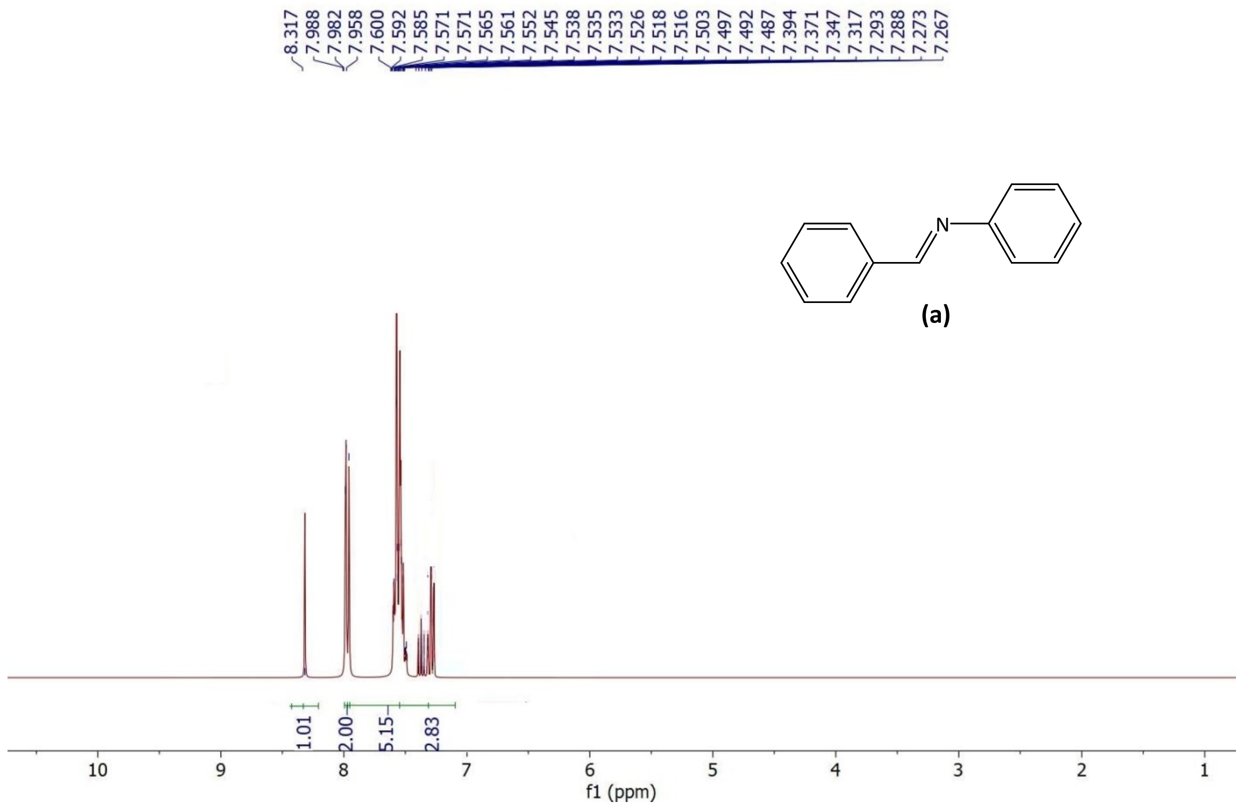

^{1}HNMR spectrum of (E)-N,1-diphenylmethanimine

^{1}H NMR (300 MHz, $CDCl_3$): δ [ppm] = 8.31 [s, 1H], 7.98-7.95 [m, 2H], 7.60-7.48 [m, 5H], 7.37-7.26 [m, 3H].

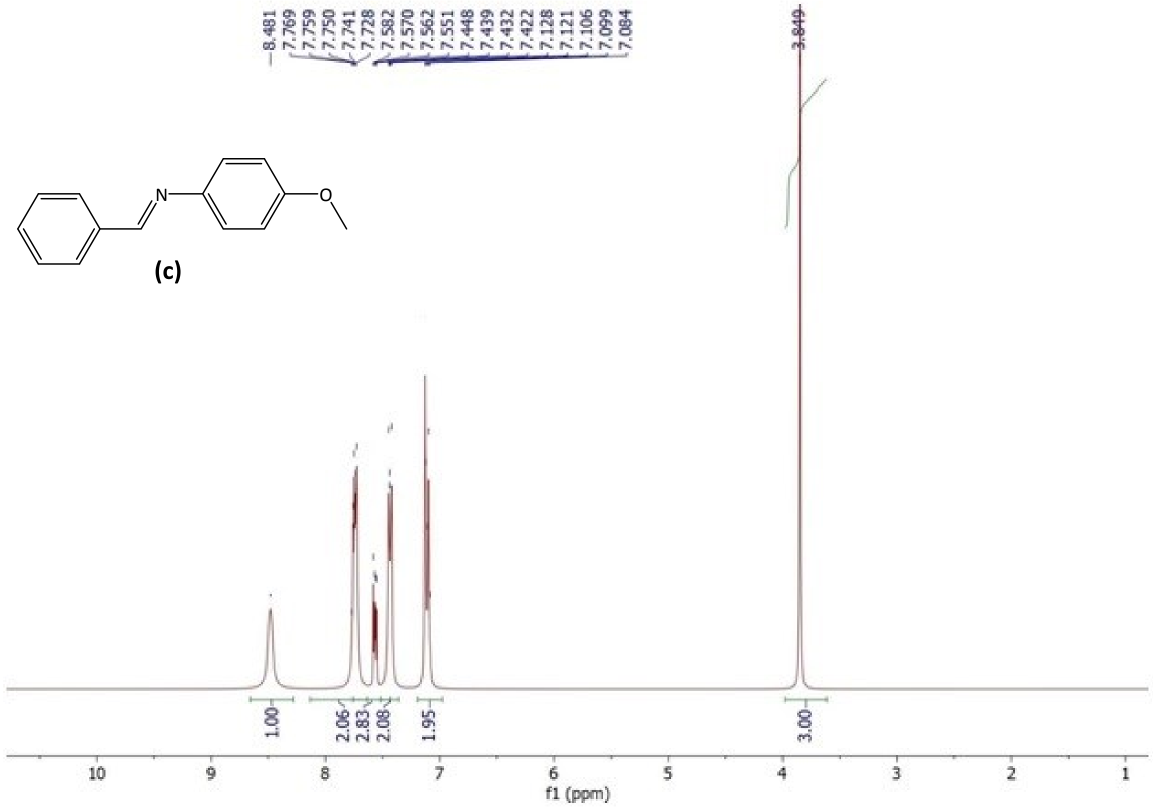

^{1}HNMR spectrum of (E)-N-(4-methoxyphenyl)-1-phenylmethanimine

^{1}H NMR (300 MHz, $CDCl_3$): δ [ppm] = 8.48 [s, 1H], 7.72-7.76 [m, 2H], 7.58-7.55 [m, 3H], 7.44-7.42 [m, 2H], 7.12-7.08 [m, 2H], 3.84 [s, 3H].

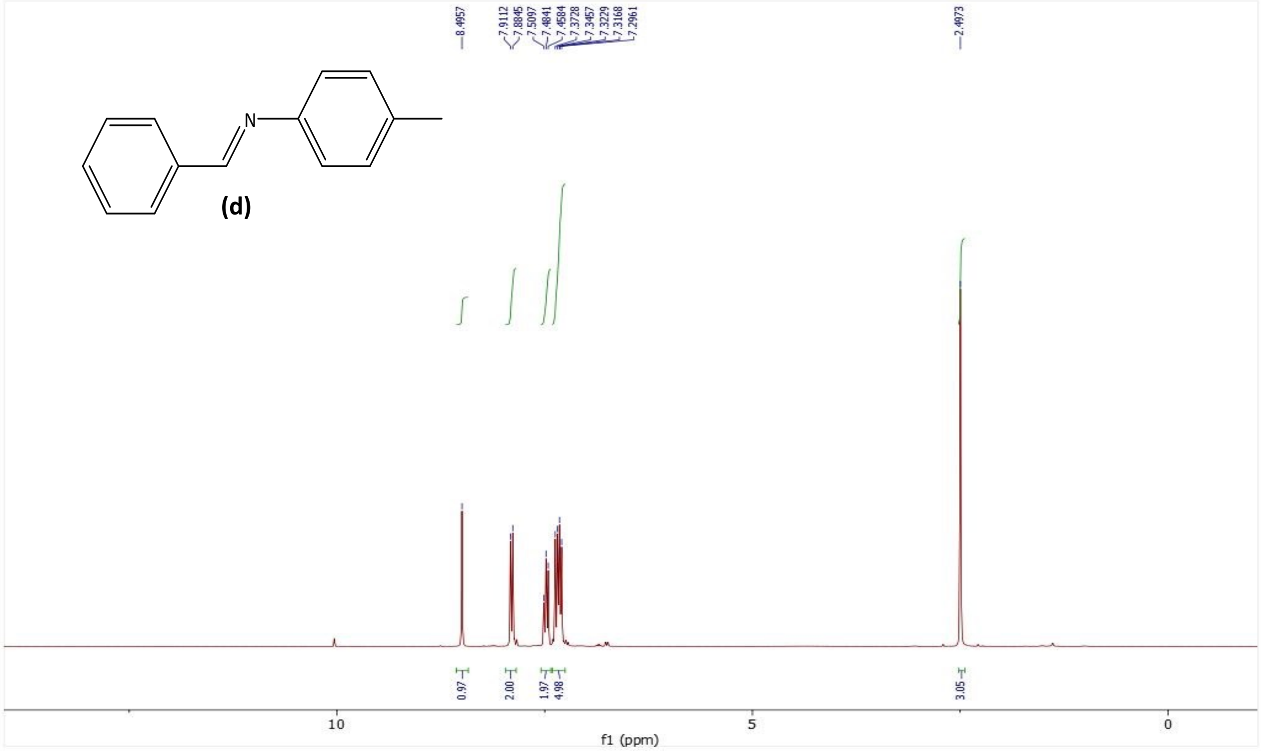

^{1}HNMR spectrum of (E)-1-phenyl-N-(p-tolyl)methanimine

^{1}H NMR (300 MHz, $CDCl_3$): δ [ppm] = 8.49 [s, 1H], 7.89 [d, *J* = 8.1 Hz, 2H], 7.50-7.45 [m, 2H], 7.37-7.29 [m, 5H], 2.49 [s, 3H].

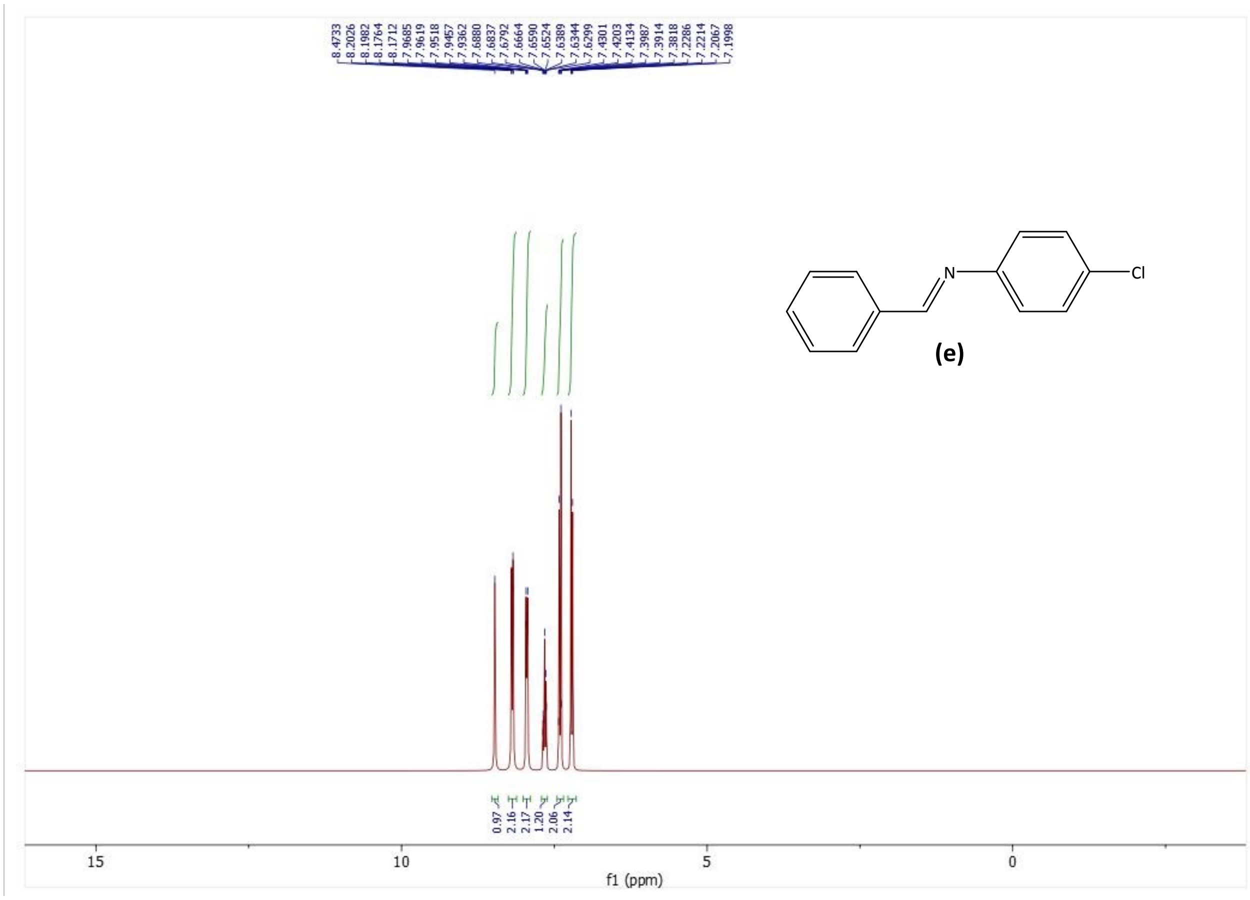

^{1}HNMR spectrum of (E)-N-(4-chlorophenyl)-1-phenylmethanimine

^{1}H NMR (300 MHz, $CDCl_3$): δ [ppm] = 8.47 [s, 1H], 8.20-8.17 [m, 2H], 7.96-7.93 [m, 2H], 7.68-7.62 [m, 1H], 7.43-7.38 [m, 2H], 7.27-7.19 [m, 2H].

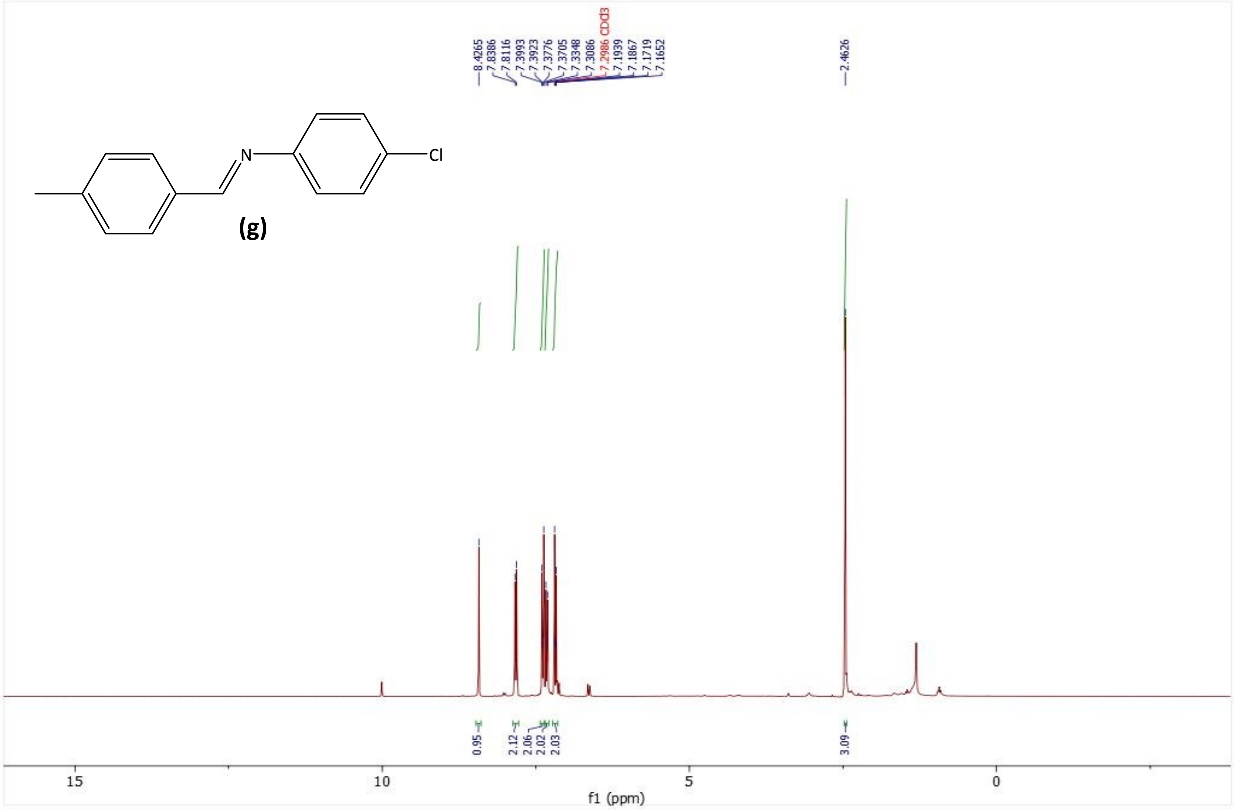

^{1}HNMR spectrum of (E)-N-(4-chlorophenyl)-1-(p-tolyl)methanimine

^{1}H NMR (300 MHz, $CDCl_3$): δ [ppm] = 8.42 [s, 1H], 7.82 [d, *J* = 8.1 Hz, 2H], 7.39-7.37 [m, 2H], 7.33-7.30 [m, 2H], 7.19-7.16 [m, 2H], 2.46 [s, 3H].

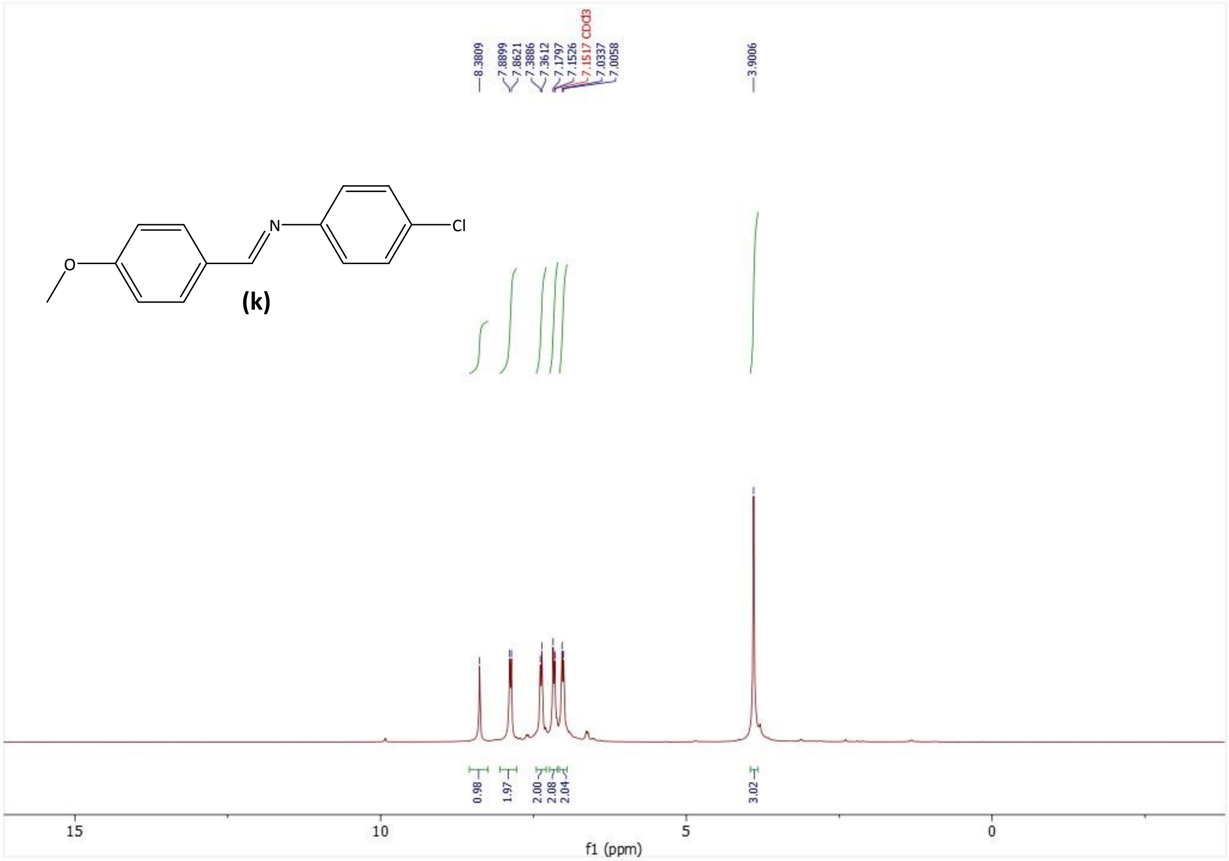

^{1}HNMR spectrum of (E)-N-(4-chlorophenyl)-1-(4-methoxyphenyl)methanimine

^{1}H NMR (300 MHz, $CDCl_3$): δ [ppm] = 8.38 [s, 1H], 7.87 [d, *J* = 8.1 Hz, 2H], 7.37 [d, *J* = 8.1 Hz, 2H], 7.16 [d, *J* = 8.1 Hz, 2H], 7.01 [d, *J* = 8.4, 2H], 3.90 [s, 3H].

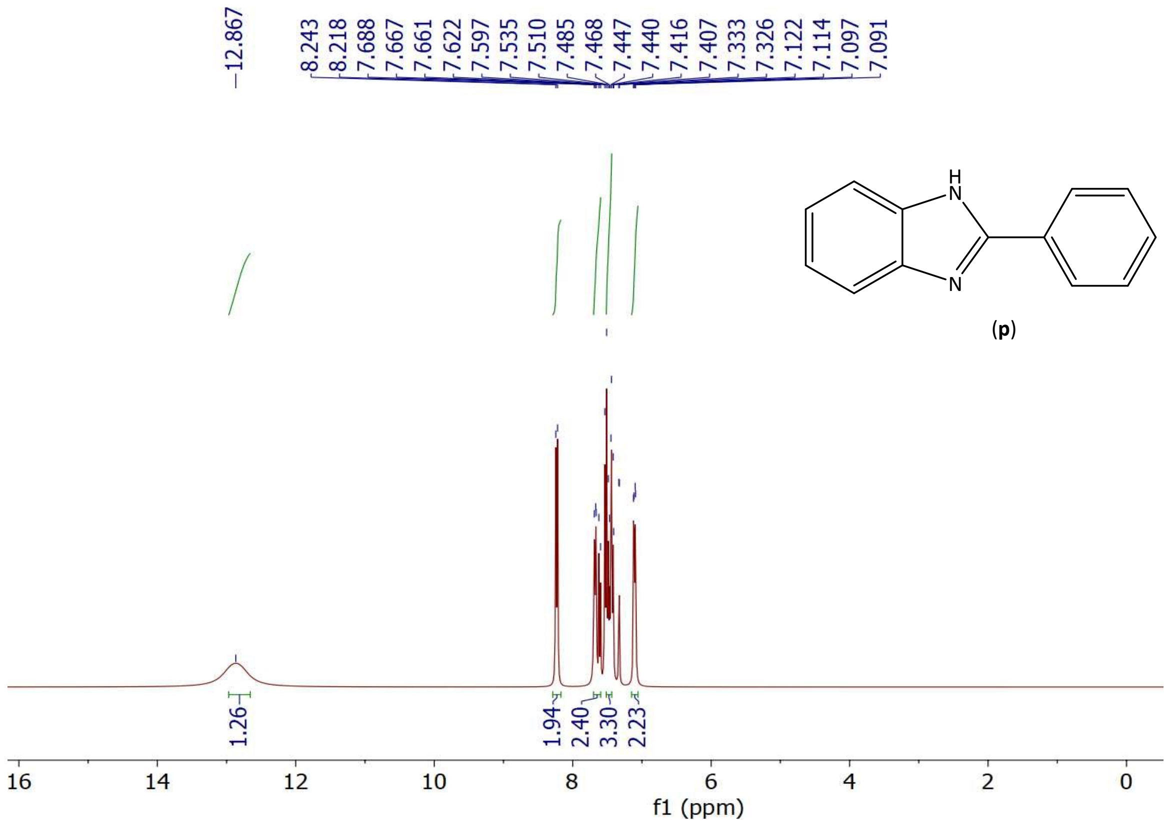

^{1}HNMR spectrum of 2-phenyl-1H-benzo[*d*]imidazole

^{1}H NMR (300 MHz, $CDCl_3$): δ [ppm] = 12.86 [s, 1H], 8.22 [d, *J* = 7.5 Hz, 2H], 7.68-7.51 [m, 2H], 7.48-7.32 [m, 3H], 7.12-7.09 [m, 2H].

Reference

1. S. Sobhani, S. Asadi, M. Salimi and F. Zarifi, J. *Organomet. Chem*., 2016, **822**, 154-164.
2. S. Sobhani and F. Zarifi, *Chin. J. Catal*., 2015, **36**, 555-563.
3. C. Grundke and T. Opatz, *Green Chem*., 2019, **21**, 2362.
4. S. Sobhani, F. O. Chahkamali and J. M. Sansano, *RSC Adv*., 2019, **9**, 1362.
5. Q. Jiang, J. –Y. Wang and C. Guo, *J. Org. Chem*., 2014, **79**, 8768.
6. K. Tanaka and R. Shraishi, *Green Chem*., 2000, **2**, 272.
7. Y. Motoyama, M. Taguchi, N. Desmira, S. –H. Yoon, I. Mochida and H. Nagashima, *Chem. Asian J*., 2014, **9**, 71.
8. D. Tan, H. Li, D. J. Young and J. Long, *Tetrahedron*, 2016, **72**, 4169.
9. M. G. Dekamin, M. Azimoshan and L. Ramezani, *Green Chem*., 2013, **1**5, 811.
10. R. Suresh, D. Kamalakkannan, K. Ranganathan, R. Arulkumaran, R. Sundararajan, S. P. Sakthinathan, S. Vijayakumar, K. Sathiyamoorthi, V. Mala, G. Vanangamudi, K. Thirumurthy, P. Mayavel and G. Thirunarayanan, *Spectrochim. Acta A. Mol. Biomol. Spectrosc*., 2013, **101**, 239.
11. R. Trivedi, S. K. De, R. A. Gibbs*, J. Mol. Catal. A. Chem.* 2006, **245**, 8.
